# Enhancing emergency medical education and training: Performance under pressure 

**DOI:** 10.3205/zma001767

**Published:** 2025-09-15

**Authors:** Nadja Spitznagel, Benjamin Gordon, Stephan Hearns, Dominik Hinzmann, Patrick Meybohm, Oliver Happel, Carlos Hölzing

**Affiliations:** 1 Ingolstadt Hospital, Clinic for Anesthesia and Intensive Care Medicine, Palliative and Pain Medicine, Ingolstadt, Germany; 2 University Hospital Würzburg, Department of Anaesthesiology, Intensive Care, Emergency and Pain Medicine, Würzburg, Germany; 3 Emergency Medical Retrieval Service (West), ScotSTAR, Paisley, Scottland; 4 Technical University of Munich, TUM School of Medicine and Health, Department of Clinical Medicine, Department of Anesthesiology and Intensive Care, Munich, Germany

**Keywords:** emergency medicine, stress management, continuing education, performance under pressure, blended learning, cognitive tools, decision making, mental training

## Abstract

Continuous development of professional skills is particularly relevant for emergency physicians because of the daily stress and high-pressure working conditions they face. Nevertheless, workshops that provide practical exercises for better handling these working conditions, especially for emergency physicians, are rare. The present workshop was designed as a part of a pilot project with a blended-learning approach. It lasted for 10 hours and took place over 1.5 days. Twenty-five emergency physicians took part: 22 from anesthesia and one participant each from trauma surgery, general surgery, and radiology. Both before and after the workshop, the participants were asked to assess and describe their experiences with this and similar workshops. The pre-workshop survey revealed that all the emergency physicians had already been in situations with high psychological pressure. They rated the psychological stress as correspondingly high. According to the participants, improving their ability to deal with stress could enhance their work performance as emergency physicians. The results of the post-workshop survey indicate that the participants felt that they had benefited from the workshop and the techniques they had learned for coping with psychological pressure in emergency situations and were able to apply these in clinical practice. This and comparable teaching formats for managing the stress of emergency physicians may prove beneficial.

## Abstract (de)

Die kontinuierliche Förderung von berufsrelevanten Fähigkeiten ist aufgrund der täglichen Belastung und dem Arbeiten unter hohem Druck für Notfallmediziner von besonderer Relevanz. Dennoch sind Workshops, die praktische Übungen für einen besseren Umgang mit diesen Arbeitsbedingungen speziell für Notfallmediziner vermitteln, selten. Wir präsentieren hier einen Workshop, der im Rahmen eines Pilotprojekts mit einem Blended-Learning-Ansatz konzipiert wurde. Die Veranstaltung umfasste 10 Stunden und fand über 1,5 Tage statt. Insgesamt nahmen 25 Notfallmediziner daran teil, darunter 22 aus der Anästhesie sowie jeweils ein Teilnehmer aus der Unfallchirurgie, Allgemeinchirurgie und Radiologie. Vor und nach dem Workshop wurden die Teilnehmenden zu ihren Einschätzungen und Erfahrungen mit diesem und ähnlichen Workshops befragt. Die Befragung vor dem Workshop ergab, dass sich alle befragten Akutmediziner bereits in Situationen mit hohem psychischem Druck befanden und die psychische Belastung als entsprechend stark bewerteten. Nach Einschätzung der Teilnehmenden könnte eine Verbesserung ihrer Fähigkeiten im Umgang mit Stress ihre Arbeitsleistung als Notfallmediziner erhöhen. Die Ergebnisse der Befragung nach dem Workshop verdeutlichen, dass die Teilnehmenden das Gefühl hatten, von dem Workshop und den darin erlernten Techniken zur Bewältigung von psychischem Druck in Notfallsituationen zu profitieren und diese in der klinischen Praxis anwenden zu können. Es konnte gezeigt werden, dass dieser und vergleichbare Lehrformate zur Stressbewältigung von Akutmedizinern einen Nutzen haben.

## 1. Context and objective

**Figure 1:**
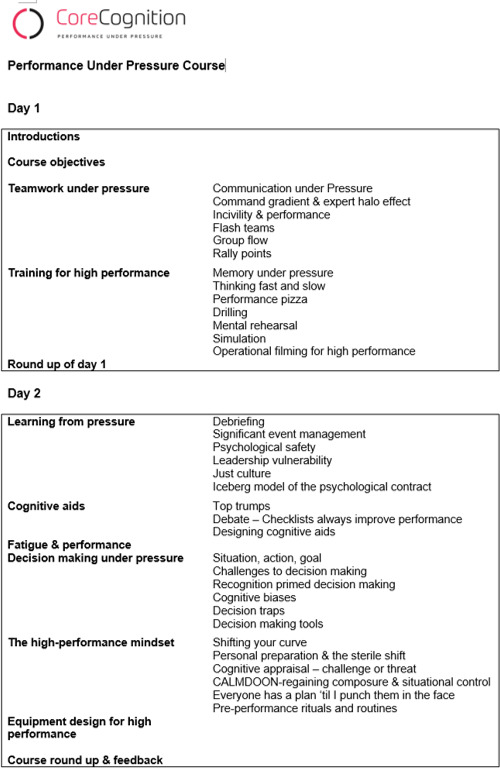
Workshop program

Continuing education and training is essential for medical professionals. In particular, emergency physicians are constantly exposed to stressful situations in their working environment, which requires them to act quickly and correctly. These situations represent a constant physical and psychological burden that must be dealt with in everyday working life. Current training courses often focus on technical skills and their further development. However, to meet the specific requirements of one’s profession, it is necessary to offer supplementary courses and supplement existing ones [1], [2], [3]. With this in mind, a workshop was designed to better prepare emergency physicians for their daily challenges and to deal with pressure situations. In terms of content, the course includes strategies for optimizing individual and organizational performance. Various aspects are covered, including the psychology of stress and decision-making, the influence of fatigue and environmental factors, and how cognitive biases can be managed. The workshop also shows how organizations can foster high-performance cultures through targeted leadership, corporate culture, and team selection. Practical techniques for coping with pressure include communication strategies, cognitive tools such as checklists, and measures for effective teamwork. Another focus is on targeted preparation through mental training (see figure 1 (Fig. 1)).

## 2. Implementation

The present workshop is based on the theoretical principles and concepts from the book “emergency mind” by Dan Dworkis and the “core cognition” approach by Dr. Stephen Hearns. The workshop was developed and offered as a 1.5-day (10 hour) event using a blended-learning concept. It included theoretical foundations as well as group work and practical exercises on the physiological effects of pressure on clinical performance, effective communication in stressful situations, critical decision-making, maintaining one’s calm and composure, and the use and development of cognitive tools. The structure and content of the curriculum are shown in figure 1 (Fig. 1). Specifically, the aim is to understand how stress and pressure affect performance and then to learn techniques for maintaining composure and control in critical situations. In addition, the development and reinforcement of core values and an understanding of cognitive bias are taught in order to improve performance and the approach to handling errors.

## 3. Evaluation

The evaluation was carried out using a two-part questionnaire. The first part of the questionnaire was completed after the course materials had been sent out and before the start of the first learning unit in the seminar room. The second part was completed in the seminar room after completing the last learning unit. The questionnaire consists of a general part (A) and a specific part (B). The questions were intended to record the expectations from the workshop, personal experiences with psychological pressure in emergency situations, and the resulting stress and experiences with previous training measures before the start of the workshop. After completion of the workshop, feedback on the experience with the course content, satisfaction and subjectively perceived improvement in skills was recorded.

## 4. Survey results

### 4.1. Survey results before the workshop

The results of the pre-workshop survey are summarized in table 1 (Tab. 1). All participants stated that improving their stress management skills would positively affect their work performance as emergency physicians. In response to the question “How often have you experienced situations involving high psychological pressure in your professional practice as an emergency physician?” 10% of respondents answered “occasionally”; 60% answered, “frequently”; and 30% answered, “very frequently” (5-point Likert scale: 1=never, 5=very frequently). In rating the stress caused by such stressful emergency situations, participants averaged a score of 7.9 ± 0.9 points (median=8; scale: 1=somewhat stressful, 10=severely stressful). In rating their own preparation for stressful emergency situations, they averaged a score of 6.1±1.8 points (median=6; scale: 1=not prepared at all, 10=very well prepared). In assessing their own ability to make clear decisions under pressure, participants averaged a score of 6.6±1.7 points (median=7; scale: 1=very low, 10=very high). They averaged 9.4±0.9 points (median=10; scale: 1=very low, 10=very open) in rating their openness to new techniques and strategies for coping with stress. Overall, 70% of respondents had already expressed a desire to take part in a workshop on coping with stress in emergency situations. When asked which aspect of dealing with stress in emergency situations should be improved the most in the workshop, 60% of participants responded that they considered the ability to maintain their calm and composure in stressful situations to be the most important aspect.

### 4.2. Survey results after the workshop

**Figure 2:**
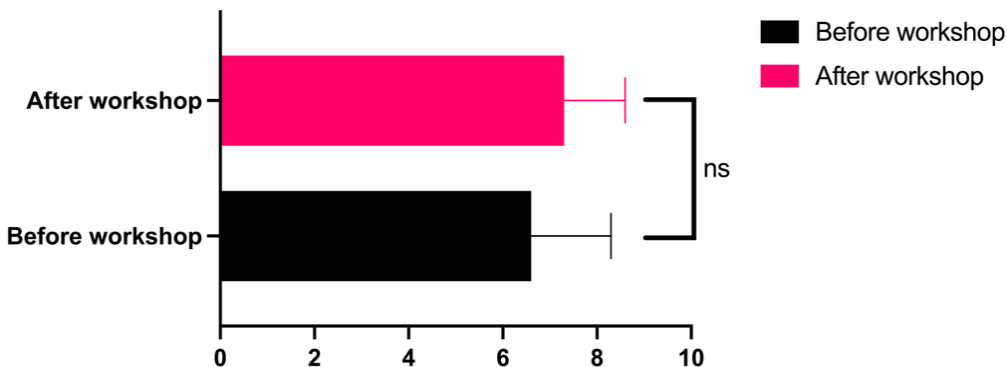
Ability to make clear decisions under pressure (1=very low, 10=very high)

Table 2 (Tab. 2) shows the results of the post-workshop survey. In assessing the improvement in their ability to make clear decisions under pressure, participants scored an average of 6.2±2.0 (median=6.5). Their overall assessment of the workshop averaged 8.1±1.6 (scale: 10=excellent). In responding to the question “How useful did you find the workshop?” (1=not at all useful, 10=extremely useful), participants assigned an average rating of 8.5±1.7. A total of 85% of respondents stated that the strategies taught in the workshop helped or greatly helped them cope better with stressful emergency situations. All participants stated that they were likely to apply the strategies learned in the workshop in their professional practice as emergency physicians. In a post-workshop assessment of their own ability to make clear decisions under pressure, participants scored an average of 7.3±1.3 (median=7; scale: 10=very high), indicating an improvement compared with pre-workshop assessments, even if the difference was not statistically significant (see figure 2 (Fig. 2)).

## 5. Outlook

This pilot project underlines the interest of emergency physicians in learning techniques and strategies for coping with stress in emergency situations. The results of the post-workshop survey show that participants felt they had benefited from the workshop and the techniques they learned to manage psychological pressure in emergency situations and apply them in clinical practice. Existing CRM/TRM course formats already provide important foundations for working in high-pressure situations. The course described here specifically expands this approach to include individual, personality-oriented components that emergency physicians need in order to remain capable of acting even in extremely demanding situations. Future studies need to demonstrate the benefits of this workshop as a part of the training curriculum for emergency physicians at scale.

## Authors’ ORCIDs

- Nadja Spitznagel: [0000-0002-3139-8977]
- Dominik Hinzmann: [0000-0001-5943-352X]
- Patrick Meybohm: [0000-0002-2666-8696]
- Oliver Happel: [0000-0001-7320-8521]
- Carlos Hölzing: [0000-0002-3732-6544]

## Competing interests

The authors declare that they have no competing interests. 

## Figures and Tables

**Table 1:**
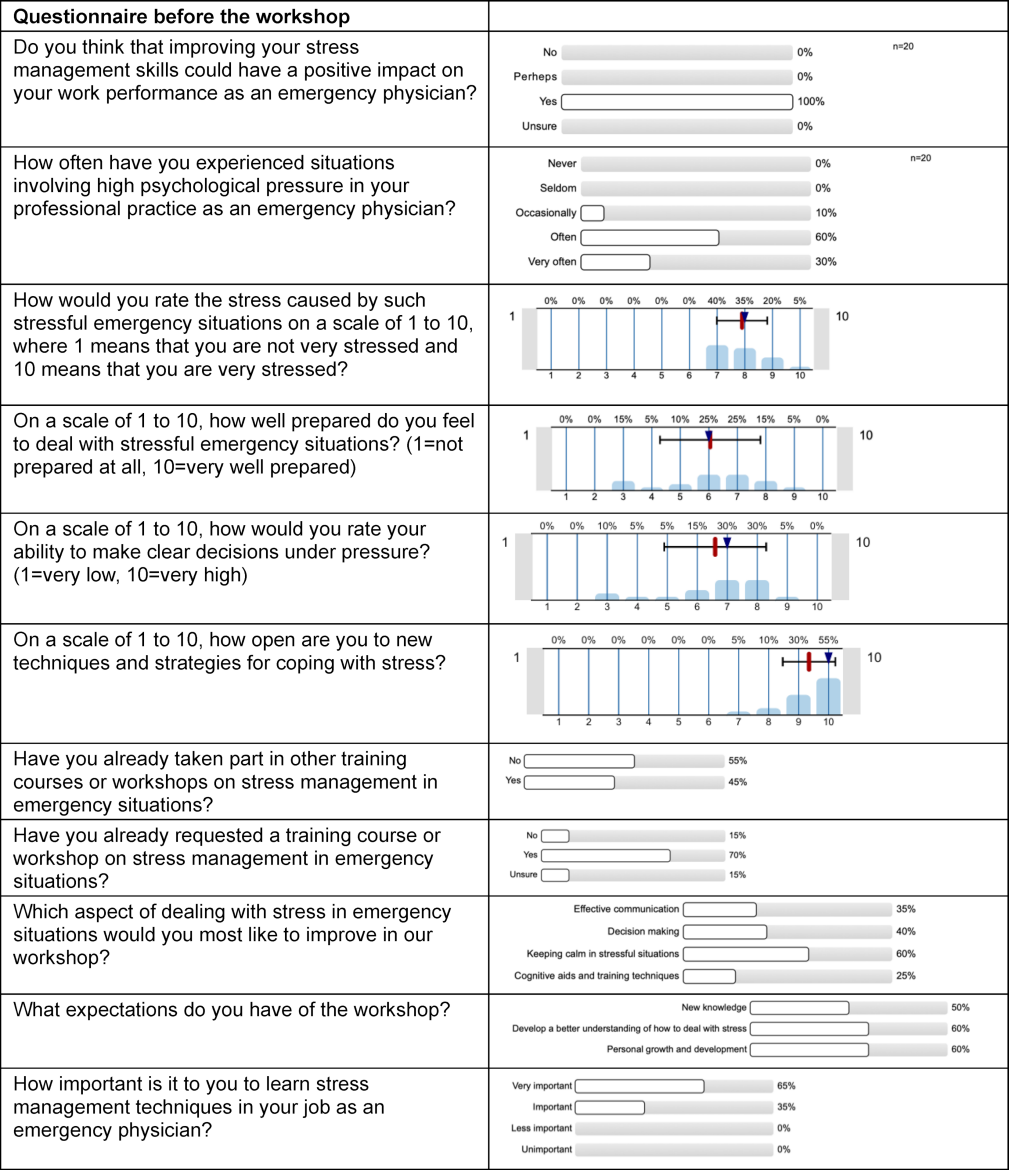
Survey results prior to the workshop

**Table 2:**
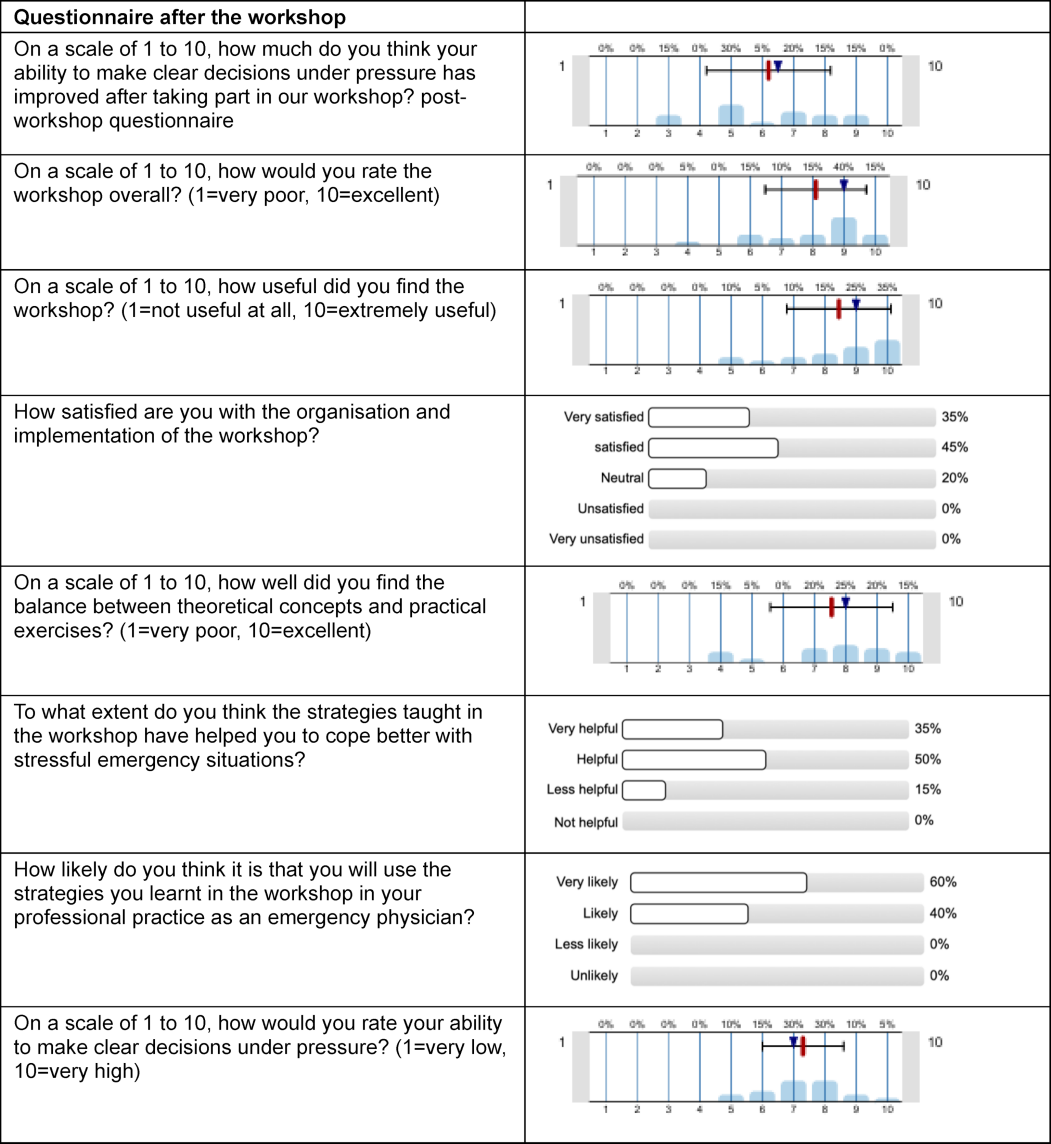
Survey results after the workshop
